# Dietary processed former foodstuffs for broilers: impacts on growth performance, digestibility, hematobiochemical profiles and liver gene abundance

**DOI:** 10.1186/s40104-024-01081-w

**Published:** 2024-09-08

**Authors:** Karthika Srikanthithasan, Marta Gariglio, Elena Diaz Vicuna, Edoardo Fiorilla, Barbara Miniscalco, Valeria Zambotto, Eleonora Erika Cappone, Nadia Stoppani, Dominga Soglia, Federica Raspa, Joana Nery, Andrea Giorgino, Roser Sala, Andrés Luis Martínez Marínz, Josefa Madrid Sanchez, Achille Schiavone, Claudio Forte

**Affiliations:** 1 Department of Veterinary Sciences, University of Turin, Grugliasco, Italy; 2 Animal Nutrition and Welfare Service (SNiBA), Department of Animal and Food Sciences, Universitat Autònoma de Barcelona, Barcelona, Spain; 3 Departamento de Producción Animal, Universidad de Córdoba, Córdoba, Spain; 4 Department of Animal Production, University of Murcia, Murcia, Spain

**Keywords:** Alternative feed, Broiler chicken, Digestibility, Former foodstuff, Gene abundance, Growth performance

## Abstract

**Background:**

The present experiment aimed to evaluate the effects of commercially processed former foodstuffs (cFF) as dietary substitutes of corn, soybean meal and soybean oil on the growth performance, apparent total tract digestibility (ATTD), hematobiochemical profiles, and liver gene abundance in broiler chickens. Two hundred one-day-old male ROSS-308 chicks were assigned to 4 dietary groups (5 replicates of ten birds per replicate) according to their average body weight (BW, 38.0 ± 0.11 g). All groups received a two-phase feeding program: starter, d 1–12 and grower, d 12–33. The control group (cFF0) was fed a standard commercial feed based on corn, soybean meal and soybean oil. The other three groups received diets in which the feed based on corn, soybean meal, and soybean oil was partially replaced with cFF at a substitution level of 6.25% (cFF6.25), 12.5% (cFF12.5) or 25% (cFF25) for the following 33 d.

**Results:**

The growth performance data showed no differences in BW or average daily gain among groups, although the average daily feed intake decreased during the grower period (12–33 d) and over entire experimental period (1–33 d) in a linear manner as the cFF inclusion level rose (*P* = 0.026), positively affecting the gain to feed ratio (*P* = 0.001). The ATTD of dry matter of the cFF-fed groups were greater with respect to control group and increased throughout the experimental period, whereas the ATTD of ether extract linearly decreased with increasing levels of cFF-fed groups compared with control group and throughout the experimental period (*P* < 0.05). Additionally, a linear increase in the heterophil to lymphocyte ratio, serum cholesterol, triglycerides and alanine-aminotransferase were observed with increasing dietary levels of cFF (*P* < 0.05); however, no differences were observed in lipoprotein lipase or sterol regulatory element binding transcription factor gene abundance.

**Conclusions:**

The results of this experiment demonstrate that it is possible to incorporate cFF into nutritionally balanced diets for broiler chickens, even up to 25% substitution levels, for up to 33 d without adversely impacting the overall growth performance of male broiler chickens raised under commercial conditions. Further studies are essential to validate the hematological trait findings.

**Supplementary Information:**

The online version contains supplementary material available at 10.1186/s40104-024-01081-w.

## Background

As poultry diets are generally grain-based, the competition for resources between feed and food is of growing concern [1]. Ongoing research strives to identity sustainable alternatives to corn and soybean meal to replace their use in monogastric animal feed [2]. In 2022, the European Commission endorsed the use of former foodstuffs in livestock feed, defining them in the catalogue of feed materials [3] as: “*foodstuffs, other than catering reflux, which were manufactured for human consumption in full compliance with the EU food law, but which are no longer intended for human consumption for practical or logistical reasons or due to problems of manufacturing or packaging defects or other defects and which do not present any health risks when used as feed.*” Europe processes approximately 5 million tons of former foodstuffs annually [4]. These materials, which are legally distinct from food waste, offer a potential means to reduce the consumption of natural resources, such as water, reducing the carbon footprint and land usage of feed production [5].

In the last decade, former foodstuffs have been referred to in the literature as ‘ex food [6]’, ‘food leftovers [7]’, ‘former food products [8]’, ‘bakery by-products [9]’, and ‘bakery meal [10]’. With advancements in industrial processes, these former foodstuffs emerged as commercially processed former foodstuffs (cFF). They are composed of a mixture of different raw materials obtained from intermediate, unfinished, and incorrect products, primarily from the bakery, confectionary and food industries. These materials undergo unpacking, mixing, grinding, and drying to become feed ingredients as a commercialised product and available on the market under Commission Regulation (EU) 2022/1104 [3]. These cFF have a high energy content due to the presence of sugar, starch, oil, and fat [8, 11, 12]. The inclusion of cFF into animal feeds has proven to reduce food waste accumulation and dependency on traded feed [5, 13].

Existing studies have shown that substituting traditional poultry feed components with cFF did not adversely affect broiler growth performance but decreased ileal digestible energy [14, 15]. It is acknowledged that differences in source materials and processing techniques can lead to variations in the chemical composition and energy content of cFF [16, 17]. Therefore, the involvement of an intermediate processor of former foodstuffs, able to standardize their composition and formulation as commercial feed, is crucial to ensure consistency among batches and provide a high-quality feed ingredient [12].

Despite the commercial availability of cFF and its recent standardization within the European legal framework, a substantial knowledge gap persists in the literature concerning its inclusion in poultry nutrition and its impact on growth performance, digestibility, liver gene abundance, and health [18]. Thus, the primary hypothesis of this experiment is that cFF, given its standardization as feed, can substitute traditional ingredients such as corn, soybean meal, and soybean oil in the broiler diet. Therefore, this experiment aimed to assess the impact of different dietary inclusion levels of cFF as a substitute for corn, soybean meal, and soybean oil in broiler diets, focusing on growth performance, apparent total tract digestibility (ATTD), hematobiochemical profiles, and liver gene abundance related to lipid metabolism.

## Materials and methods

### Bird management and experimental diets

This experiment was conducted at the poultry facility of the University of Turin (North-West Italy). The experimental protocol was approved by the Bioethical Committee of the Department of Veterinary Sciences, University of Turin, Italy (protocol no. 245, 01/01/2022). A total of 200 one-day-old male Ross-308 broiler chicks were used in the 33-d experimental period. Chicks were individually weighed and divided into 4 dietary groups based on their initial body weight (BW; 38.0 ± 0.11 g, on average), each group comprised 5 pens of 10 chicks per pen. Each pen measured 1 m^2^ and had an automatic ventilation system, rice hull litter, individual feeders and drinkers. For the initial three weeks, infrared lamps were used to maintain the temperature recommended for standard breeding practices, and the lighting schedule followed the Aviagen guidelines [19]. Chicks received vaccinations for Newcastle disease, Gumboro disease, infectious bronchitis and coccidiosis upon hatching. The birds and their environmental parameters were checked daily throughout the experimental period.

The cFF (PRIMO^®^) used in this experiment was provided by Dalma Mangimi SPA (Cuneo, Italy). The cFF’s ingredient list can be divided into 3 main categories, listed in decreasing order of relative quantity in the finished product: bakery by-products (such as wafers, biscuits, bread, crackers, snack, croissants, cakes, seasonal traditional desserts, breadsticks, sliced bread), former foodstuffs (dry pasta, chocolate, puffed cereals), and agro-industrial by-products (cocoa nib shells and hazelnut skins). These materials are primarily sourced through large-scale retail trade and pre-selling production phases, as defined by EU regulation 2022/1104. The cFF contains no other non-food ingredients, and specific recipes, unpacking methods, processing methodologies, and mixing information are protected under the patent rights of the producer.

Based on the cFF chemical composition (Table 1), four experimental diets were formulated for the two distinct feeding phases: starter (from d 1 to 12) and grower (from d 13 to 33), as shown in Table 2. These diets were intended to meet the nutritional needs of broilers with nitrogen-corrected apparent metabolizable energy (AMEn, calculated) levels set at 3,000 kcal/kg for starter and 3,100 kcal/kg for grower phases, in accordance with National Research Council guidelines [20]. The control group received corn, soybean meal and soybean oil based standard commercial feed (cFF0). The other three experimental diets incorporated the cFF ingredient, substituting corn, soybean meal and soybean oil at the following percentages w/w: 6.25% (cFF6.25), 12.5% (cFF12.5), and 25% (cFF25) (Table 2). All diets were administered in crumble form (pelleting temperature 60 °C, size 0.4 cm, humidity 12%). Feed and water were provided ad libitum.

**Table 1:** Chemical composition of the commercially processed former foodstuffs (cFF) used in experimental diets^a^

**Chemical composition, % as fed**	**cFF**
DM	88
CP	10.8
EE	9.0
CF	1.9
NFE	64.4
Ash	1.9
Starch	41
Total sugar^b^	18
Sodium	0.35
Calcium	0.13
Phosphorus	0.18
Potassium	0.23
Chloride	0.49
Amino acids, % in CP	
Lysine	0.24
Cystine	0.20
Phenylalanine	0.51
Isoleucine	0.37
Leucine	0.71
Methionine	0.16
Valine	0.47
Tyrosine	0.31
Proline	1.07
Alanine	0.34
Threonine	0.30
Arginine	0.39
Histidine	0.22
Glycine	0.37
Serine	0.48
Glutamic acid	3.27
Aspartic acid	0.52
Fatty acids, % in total FA	
ΣSFA	39.76
ΣMUFA	46.16
ΣPUFA	14.08

*cFF* Commercially processed former foodstuffs, *DM* Dry matter*, CP* crude protein*, CF* Crude fiber*, EE* Ether extract, *NFE* Nitrogen free extract*, FA* Fatty acids, *ΣSFA* Total saturated fatty acids*, ΣMUFA* Total monounsaturated fatty acids*, ΣPUFA* Total polyunsaturated fatty acids

^a^The commercially processed former foodstuffs (PRIMO^®^) used in this experiment was provided by Dalma Mangimi SPA (Cuneo, Italy)

^b^Expressed in sucrose

**Table 2:** Ingredients (g/kg as fed) and analysed chemical composition of the experimental diets^a^

Diet composition	Starter (d 1–12)				Grower (d 12–33)			
	**cFF0**	**cFF6.25**	**cFF12.5**	**cFF25**	**cFF0**	**cFF6.25**	**cFF12.5**	**cFF25**
Corn meal	498.5	452	409	314.5	526.5	482	434.5	342.5
Soybean meal	397	390.5	381	369	359	350.5	345	331
cFF	-	62.5	125	250	-	62.5	125	250
Soybean oil	58.5	49	39	20.5	69.5	60	50.5	31.5
Dicalcium phosphate	5.0	5.0	5.0	5.0	5.0	5.0	5.0	5.0
Calcium carbonate	17.7	17.7	17.7	17.7	15.8	15.8	15.8	15.8
Sodium chloride	1.5	1.5	1.5	1.5	2.9	2.9	2.9	2.9
Sodium bicarbonate	3.2	3.2	3.2	3.2	3.5	3.5	3.5	3.5
DL-Methionine	4.5	4.5	4.5	4.5	4.5	4.5	4.5	4.5
L-Lysine	2.3	2.3	2.3	2.3	1.5	1.5	1.5	1.5
Vitamin-mineral premix^b^	4.7	4.7	4.7	4.7	4.7	4.7	4.7	4.7
Choline chloride	0.1	0.1	0.1	0.1	0.1	0.1	0.1	0.1
Optifos 250 bro	1.0	1.0	1.0	1.0	1.0	1.0	1.0	1.0
Avizyme 1,500x	1.0	1.0	1.0	1.0	1.0	1.0	1.0	1.0
Titanium dioxide	5.0	5.0	5.0	5.0	5.0	5.0	5.0	5.0
Total	1,000	1,000	1,000	1,000	1,000	1,000	1,000	1,000
AMEn^c^, kcal/kg	3,000	3,000	3,000	3,001	3,100	3,101	3,100	3,100
Chemical composition^d^ (analysed)								
DM^e^, %	91.3	91.6	91.2	92.0	90.9	90.9	90.4	91.6
CP^e^, %	23.5	23.9	23.1	23.6	21.0	20.8	20.9	20.6
EE^e^, %	7.41	6.92	6.89	6.12	9.27	8.83	8.01	7.37
CF^e^, %	1.78	1.50	1.65	1.21	1.87	1.58	1.69	1.57
Ash^e^, %	6.31	6.66	6.42	6.66	6.28	6.12	5.82	6.35
Total sugar^f,g^, %	3.85	4.86	5.84	7.84	3.61	4.60	5.60	7.60
Starch^g^, %	31.95	31.54	31.34	30.41	33.75	33.46	32.98	32.20
Fatty acid composition, % of total FA								
SFA	18.34	23.33	22.92	28.49	17.87	20.21	21.67	26.97
MUFA	23.20	27.34	27.25	31.91	23.12	24.65	27.85	32.64
PUFA	58.46	49.45	49.94	39.60	59.00	55.14	50.49	40.49
PUFA/SFA	3.19	2.12	2.18	1.39	3.30	2.73	2.33	1.50
Σn-6	52.75	44.86	45.29	36.27	53.09	49.74	45.87	37.14
Σn-3	5.71	4.59	4.65	3.32	5.91	5.40	4.62	3.35
Σn-6/Σn-3	9.24	9.78	9.75	10.9	8.98	9.21	9.94	11.1

*AMEn* Nitrogen-corrected apparent metabolizable energy*, DM* Dry matter, *CF* Crude fiber*, CP* Crude protein*, EE* Ether extract, *FA* Fatty acids*, SFA* Saturated fatty acids*, MUFA* Monounsaturated fatty acids, *PUFA* Polyunsaturated fatty acids, *Σn-6* Total omega-6, *Σn-3* Total omega-3, *cFF* Commercially processed former foodstuffs

^a^Four dietary treatments: *cFF0* control diet (based on corn, soybean meal and soybean oil), *cFF6.25* 6.25% w/w substitution of corn, soybean meal and soybean oil with cFF, *cFF12.5* 12.5% w/w substitution of corn, soybean meal and soybean oil with cFF, *cFF25* 25% w/w substitution of corn, soybean meal and soybean oil with cFF

^b^Mineral-vitamin premix: vitamin A (retinyl acetate), 12,500 IU; vitamin D_3_ (cholecalciferol), 3,500 IU; vitamin E (DL-a-tocopheryl acetate), 40 mg; vitamin K (menadione sodium bisulfite), 2.0 mg; biotin, 0.20 mg; thiamine, 2.0 mg; riboflavin, 6.0 mg; pantothenate, 15.21 mg; niacin, 40.0 mg; choline, 750.0 mg; pyridoxine, 4.0 mg; folic acid, 0.75 mg; vitamin B_12_, 0.03 mg; Mn, 70 mg; Zn, 62.15 mg; Fe, 50.0 mg; Cu, 7.0 mg; I, 0.25 mg; Se, 0.25 mg

^c^Calculated according to INRA, 2004 [51]. AMEn of cFF as communicated by Dalma Mangimi spa, Cuneo, Italy

^d^The chemical analyses were carried out on three replicates of each feed sample

^e^% as fresh matter basis

^f^Expressed in sucrose

^g^% as fed DM

### Growth performance

At the beginning of the experiment, birds were individually labelled with a wing mark. The experimental period lasted 33 d, during which bird health status and mortality were monitored daily. The BW (g) of each bird was recorded upon its arrival and at the end of each feeding phase (1, 12, and 33 days of age, respectively). Feed intake (g) per replicate was recorded at the end of each feeding phase. The average daily gain (ADG, g/d), average daily feed intake (ADFI, g/d) and the gain to feed ratio (G:F, g/g) were calculated on the replicate basis for each feeding phase and for the whole experimental period (d 1–12, d 12–33, and d 1–33, respectively).

### Digestibility

The digestibility experiment was conducted at the end of each feeding phase. The indigestible marker titanium dioxide (TiO_2_) (5 g/kg) was added to the feed during the formulation of the experimental diets (Table 2) to evaluate the ATTD. Excreta was collected according to the methods outlined by Dabbou et al. [21]. In brief, all birds of each replicate were removed from the pens and housed in wire-mesh cages (1 cage/replicate) to collect fresh excreta samples for approximately 1 h/d for 3 consecutive days. Following the collection, the excreta samples collected from each replicate over the 3 d were pooled and frozen at –20 °C until freeze-drying and analysis.

### Feed and excreta chemical analyses

The feed, cFF ingredient, and dried excreta were analysed for dry matter (DM, 943.01), ash (942.05), crude protein (CP, 984.13), ether extract (EE, 2003.05), and crude fibre (978.10) using the standard methods outlined by the AOAC International [22, 23]. The dietary starch and total sugar were determined for the cFF ingredient and all four diets, while the mineral contents were determined only for the cFF ingredient. All analyses were performed in accordance with the method specified in (EC) No 152/2009 [24]. To determine the amino acid content of cFF, samples underwent a 22-h hydrolysis step in 6 mol/L HCl at 112 °C under a nitrogen atmosphere. The amino acids in the hydrolysate were determined by HPLC (Waters Alliance System with a Waters 1525 Binary HPLC pump, Waters 2707 autosampler, and Waters 2475 multi λ Fluorescence Detector, Milford, USA) after derivatization, following the procedure described by Madrid et al. [25].

Excreta uric acid content was determined by spectrophotometry (UNICAN UV–Vis Spectrometry, Helios Gamma, UK) in accordance with the Marquardt method [26]. All analyses were performed on two replicates per sample. The CP amount in the excreta was corrected for uric acid nitrogen. The TiO_2_ content of feed and freeze-dried excreta was assessed on a UV spectrophotometer (UNICAN UV–vis Spectrometry, Helios Gamma, UK) according to the method reported by Myers et al. [27]. The ATTD for DM, CP, and EE was calculated according to National Research Council guidelines [28].

The fatty acid profile of feed (% of total fatty acid methyl esters) was determined according to the methods described by Sukhija and Palmquist [29], and included saturated fatty acids (SFA), monounsaturated fatty acids (MUFA), polyunsaturated fatty acids (PUFA), and total n-6 and total n-3 fatty acids. PUFA/SFA and n-6/n-3 ratios were calculated.

### Hematobiochemical profiles

At the end of the experiment (d 33), three birds per replicate (*n* = 15 birds per dietary group) were slaughtered. Blood samples were collected from the jugular vein and 2.5 mL transferred into a EDTA tube and into a serum-separating tube. Blood smears were prepared from anticoagulant-free blood drops and stained using May-Grünwald and Giemsa stains [30]. Natt-Herrick solution-treated blood samples were used for total red and white blood cell counts using an improved Neubauer Hemacytometer [31]. One hundred leukocytes, comprising granular (heterophils, eosinophils, and basophils) and non-granular (lymphocytes and monocytes) types, were counted on each slide and expressed as a percentage of the total leukocytes. The heterophil to lymphocyte ratio was calculated according to Campbell [30].

Serum was obtained by allowing the anticoagulant-free tubes to stand at room temperature for approximately 2 h before centrifuging at 700 × *g* for 15 min. The resulting serum was frozen at –80 °C until further analysis. Total protein was quantified using the Biuret method (Bio Group Medical System kit), and the serum's electrophoretic pattern was obtained using a semi-automated agarose gel electrophoresis system (Sebia Hydrasys^®^). Enzymatic methods were used to measure alanine-aminotransferase, aspartate aminotransferase, gamma-glutamyl transferase, triglycerides, cholesterol, phosphorus, magnesium, iron, chloride, uric acid, and creatinine serum concentrations on a clinical chemistry analyser (Screen Master Touch, Hospitex diagnostics Srl., Firenze, Italy), as described by Salamano et al. [32].

### Liver gene abundance analysis

On the day of slaughter (d 33), liver samples (*n* = 5 per dietary group) were taken from 20 broiler chickens and stored in RNAlater at –80 °C until RNA extraction. The nine liver genes analysed, involved in lipid and stress metabolism, were as follows: acyl-CoA oxidase-1 (*ACOX1*), fatty acid binding protein-1 (FABP1), heat shock protein (*HSPA2*), caspase-6 (*CASP6*), catalase (*CAT*), fatty acid desaturase-2 (*FADS2*), lipoprotein lipase (*LPL*), superoxide dismutase-1 (*SOD1*), and sterol regulatory element binding transcription factor-2 (*SREBF2*). Additionally, beta-actin (*ACTB*) and glyceraldehyde-3-phosphate dehydrogenase (*GAPDH*) were analysed as housekeeping genes. Total RNA was extracted using the FastGene^®^ RNA Premium Kit, and its quantity (Qbit^®^, RNA Broad-Range Assay Kit) and integrity (RIN, Agilent 2,100 Bioanalyzer) analysed. Subsequently, all RNA was reverse-transcribed using the first strand cDNA synthesis kit, and cDNA was quantified using Qubit^®^. A 1:50 dilution of cDNA was used to determine the appropriate concentration. For next-generation sequencing, library was prepared by purifying the multiplex polymerase chain reaction products (ExoSAP-IT^®^ Express), followed by index polymerase chain reaction. The products of the index polymerase chain reaction were quantified by Qubit^®^ and their sizes analysed using a bioanalyzer (Agilent 2100). Each library was diluted to 4 nmol/L and pooled for next-generation sequencing on the MiSeq Illumina platform. Multiplex digital expression gene analysis was performed by MiSeq Illumina [33].

### Statistical analysis

Statistical analysis was performed using the IBM SPSS software package (version 21 for Windows, SPSS Inc., Chicago, IL, USA). Shapiro–Wilk’s test was used to establish data distributions. The assumption of equal variances was assessed using Levene’s test. Each replicate was considered an experimental unit in the evaluation of growth performance and digestibility (*n* = 5 replicates per dietary group), whereas the individual bird was used as the experimental unit for the analysis of blood parameters (*n* = 15 birds per dietary group). The collected data were analysed using one-way ANOVA. Polynomial contrasts were used to test the linear and quadratic responses to increased levels of cFF inclusion in the diet. Differences among dietary groups were considered statistically significant for *P* value ≤ 0.05. Results were expressed as means plus standard error of the mean (SEM).

R software (version 4.2.2) was used for statistical analysis of the gene abundance data. Read counts were performed using the package ‘featureCounts’; differential abundance gene analysis was conducted using the package ‘DESeq2 R’, with an adjusted *P* value < 0.05 as the threshold. Each dietary group was compared against all others to search for differential gene abundances. Differences among different experimental groups were visualised using the R package ‘Enhanced Volcano’ [34]. A principal component analysis (PCA) plot was generated to provide an overview of the differences in gene abundances among the dietary groups, visualized using R software.

## Results

### Chemical composition of experimental diets

The chemical compositions of the cFF ingredient and the four experimental diets are summarized in Tables 1 and 2, respectively. Starch content was similar among the 4 diets. As the inclusion level of cFF increased, the EE content of the experimental diets decreased, while total sugar increased. Fatty acids analysis (% of total fatty acid methyl esters) indicated a corresponding rise in SFA and MUFA content, as well as an increase in the n-6/n-3 ratio with increasing cFF inclusion level. Conversely, PUFA levels and the PUFA/SFA ratio decreased as cFF inclusion level increased.

### Growth performance

No differences among dietary groups in terms of BW and ADG were registered across the whole experimental period (Table 3). Additionally, even if ADFI and G:F showed no differences during the starter feeding phase (d 1–12), a linear decrease (*P* < 0.05) in ADFI and a linear increase (*P* < 0.05) in G:F were observed during the grower phase (d 12–33) and the overall experimental period (d 1–33) for increasing cFF inclusion levels. A lower ADFI (*P* = 0.019) was noted in cFF25 compared with the other dietary groups during the grower period (d 12–33). In relation to the overall experimental period (d 1–33), feed consumption was 8% lower in the cFF25 group compared with the control group (*P* = 0.026).

**Table 3:** The effect of different levels of cFF in the broiler diet on growth performance

Growth performance	Age	Dietary treatments				SEM	*P*-value	
		**cFF0**	**cFF6.25**	**cFF12.5**	**cFF25**		**Linear**	**Quadratic**
BW, g	1 d	38.1	38.2	38.0	38.0	0.25	0.140	0.614
	12 d	329	330	329	341	3.32	0.283	0.459
	33 d	1,977	2,015	1,961	2,041	25.54	0.569	0.702
ADG, g/d	1–12 d	26.5	26.6	26.5	27.5	0.30	0.279	0.455
	12–33 d	78.5	80.2	77.7	81.0	1.10	0.634	0.751
	1–33 d	60.6	61.8	60.1	62.6	0.80	0.568	0.701
ADFI, g/d	1–12 d	27.0	26.4	27.0	28.3	0.34	0.143	0.159
	12–33 d	129	125	125	117	1.73	0.019	0.469
	1–33 d	93.9	90.9	91.1	86.4	1.12	0.026	0.662
G:F, g/g	1-12d	0.98	1.01	0.98	0.97	0.01	0.397	0.152
	12–33 d	0.61	0.64	0.62	0.69	0.01	0.001	0.160
	1–33 d	0.65	0.68	0.66	0.73	0.01	0.001	0.183

*cFF* Commercially processed former foodstuffs, *cFF0* Control diet (based on corn, soybean meal and soybean oil), *cFF6.25* 6.25% w/w substitution of corn, soybean meal and soybean oil with cFF, *cFF12.5* 12.5% w/w substitution of corn, soybean meal and soybean oil with cFF, *cFF25* 25% w/w substitution of corn, soybean meal and soybean oil with cFF, *SEM* Standard error of the mean*, BW* Body weight*, ADG* Average daily gain, *ADFI* Average daily feed intake*, G:F* Gain to feed ratio

### Apparent total tract digestibility

The results of apparent total tract digestibility analysis are detailed in Table 4, showing no differences among dietary groups in the ATTD of CP in either feeding phase. The ATTD of EE linearly decreased (*P* < 0.05) with increasing cFF level, whereas the ATTD of DM showed a linear increase (*P* < 0.05) with increasing cFF level throughout the two feeding phases. Notably, quadratic responses (*P* < 0.05) were observed during the starter period in the ATTD of EE and during the grower period in the ATTD of DM.

**Table 4:** Effect of the different levels of cFF in broiler diets on the apparent total tract digestibility

Age	ATTD	Dietary treatments				SEM	*P*-value	
		**cFF0**	**cFF6.25**	**cFF12.5**	**cFF25**		**Linear**	**Quadratic**
1–12 d Starter	DM	0.941	0.942	0.948	0.953	0.002	0.006	0.403
	CP	0.918	0.925	0.918	0.917	0.003	0.693	0.479
	EE	0.975	0.977	0.974	0.957	0.002	0.001	0.006
12–33 d Grower	DM	0.938	0.965	0.954	0.957	0.003	0.003	0.001
	CP	0.877	0.921	0.883	0.895	0.006	0.761	0.131
	EE	0.984	0.990	0.981	0.976	0.002	0.021	0.090

*cFF* Commercially processed former foodstuffs, *cFF0* Control diet (based on corn, soybean meal and soybean oil), *cFF6.25* 6.25% w/w substitution of corn, soybean meal and soybean oil with cFF, *cFF12.5* 12.5% w/w substitution of corn, soybean meal and soybean oil with cFF, *cFF25* 25% w/w substitution of corn, soybean meal and soybean oil with cFF, *ATTD* Apparent total tract digestibility, *SEM* Standard error of the mean*, DM* Dry matter*, CP* Crude protein*, EE* Ether extract

### Hematobiochemical profiles

As outlined in Table 5, the percentage of heterophils and the heterophil to lymphocyte ratio increased linearly (*P* < 0.001) as the cFF inclusion level increased, whereas the percentage of lymphocytes underwent a linear decrease (*P* = 0.001). Dietary treatment had no impact on monocytes, eosinophils, or basophils.

**Table 5:** Effect of the different levels of cFF in broiler diets on hematobiochemical profiles

Hematological traits	Dietary treatments				SEM	*P*-value			
	**cFF0**	**cFF6.25**	**cFF12.5**	**cFF25**		**Linear**	**Quadratic**		
Heterophils, %	38.8	39.3	47.1	48.9	1.11	< 0.001		0.758	
Lymphocytes, %	43.2	43.2	37.4	34.5	1.10	0.001		0.475	
Monocytes, %	8.57	7.33	8.40	8.17	0.51	0.977		0.629	
Eosinophils, %	6.43	7.07	4.73	5.80	0.47	0.312		0.816	
Basophils, %	3.03	3.10	2.33	2.67	0.26	0.439		0.804	
H/L	0.95	0.97	1.35	1.51	0.06	< 0.001		0.540	
Serum proteins and lipids									
Proteins, g/dL	2.58	2.61	2.63	2.70	0.03	0.170			0.744
Triglycerides, mg/dL	82.7	88.5	95.6	105.8	3.59	0.018			0.752
Cholesterol, mg/dL	93.5	100	101	106	1.36	0.002			0.654
Minerals									
Phosphorus, mg/dL	3.35	3.14	3.87	3.74	0.09	0.011		0.803	
Chloride, mEq/L	95.8	97.0	101	104	0.74	< 0.001		0.412	
Iron, μg/dL	111	110	112	166	9.23	0.042		0.130	
Magnesium, mEq/L	2.09	1.95	2.09	2.07	0.02	0.697		0.198	
Liver function									
AST, IU/L	205	227	216	215	4.31	0.631		0.177	
ALT, IU/L	3.10	3.50	3.70	4.20	0.19	0.045		0.895	
GGT, IU/L	18.6	17.7	18.3	19.6	0.57	0.495		0.353	
Renal function									
Uric acid, mg/dL	3.71	3.92	3.67	4.29	0.15	0.263		0.489	
Creatinine, mg/dL	0.38	0.38	0.35	0.37	0.01	0.579		0.704	

*cFF* Commercially processed former foodstuffs*, cFF0* Control diet (based on corn, soybean meal and soybean oil), *cFF6.25* 6.25% w/w substitution of corn, soybean meal and soybean oil with cFF, *cFF12.5* 12.5% w/w substitution of corn, soybean meal and soybean oil with cFF, *cFF25* 25% w/w substitution of corn, soybean meal and soybean oil with cFF, *H/L* Heterophil to lymphocyte ratio*, SEM* Standard error of the mean*, AST* Aspartate aminotransferase*, ALT* Alanine-aminotransferase*, GGT* Gamma-glutamyl transferase

Considering the serum parameters (Table 5), we observed no differences in total protein level among dietary groups. By contrast, triglycerides and cholesterol exhibited a linear increase (*P* < 0.05) as the cFF inclusion level increased, with the highest values being observed in the cFF25 group. As for the analysis of serum minerals, iron, phosphorus, and chloride exhibited differences (*P* < 0.05) among dietary groups, whereas magnesium remained unaffected. In particular, levels of chloride and iron increased linearly (*P* < 0.05) with increasing cFF inclusion level. Indicators of liver function, including aspartate aminotransferase and gamma-glutamyl transferase, expressed no notable variations among dietary groups. Conversely, alanine-aminotransferase displayed a linear increase (*P* = 0.045) as cFF inclusion level increased, with the highest value observed in the cFF25 group. The inclusion of cFF in the diet had no effect on biomarkers of kidney function, namely creatinine and uric acid.

### Liver gene abundance

**Fig. 1:**
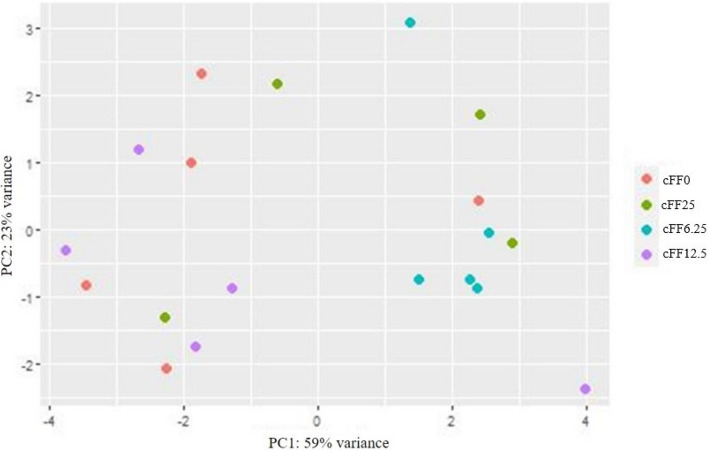
Gene abundance profiling; Principal component analysis. *PC* Principal component*, cFF* Commercially processed former foodstuffs*, cFF0* Control diet (based on corn, soybean meal and soybean oil), *cFF6.25* 6.25% w/w substitution of corn, soybean meal and soybean oil with cFF, *cFF12.5* 12.5% w/w substitution of corn, soybean meal and soybean oil with cFF, *cFF25* 25% w/w substitution of corn, soybean meal and soybean oil with cFF

**Fig. 2:**
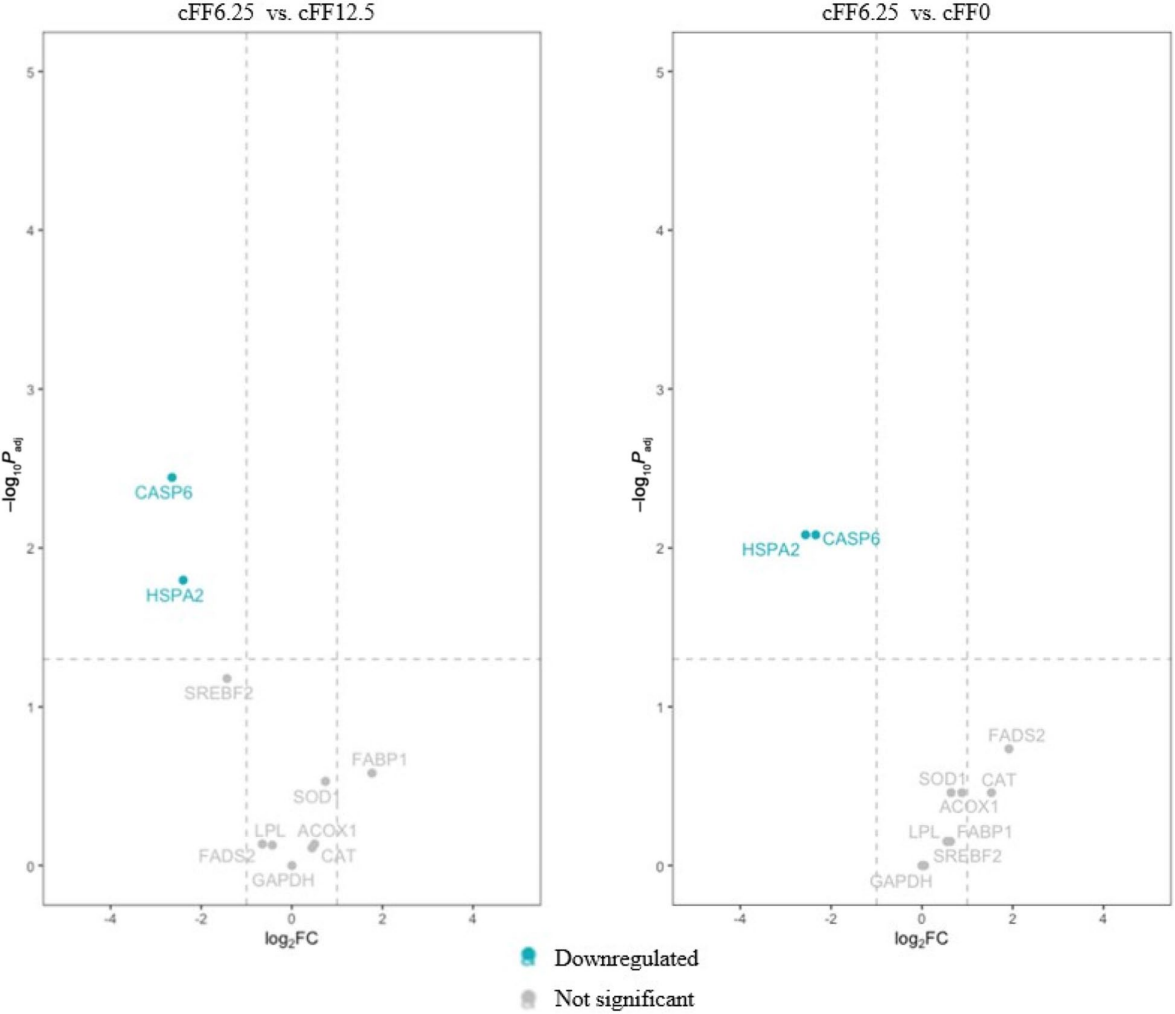
Gene abundance profiling; Volcano plot. *cFF* Commercially processed former foodstuffs*, cFF0* Control diet (based on corn, soybean meal and soybean oil), *cFF6.25* 6.25% w/w substitution of corn, soybean meal and soybean oil with cFF, *cFF12.5* 12.5% w/w substitution of corn, soybean meal and soybean oil with cFF, *cFF25* 25% w/w substitution of corn, soybean meal and soybean oil with cFF), *ACOX1* Acyl-CoA oxidase-1, *FABP1* Fatty acid binding protein-1, *HSPA2* Heat shock protein, *CASP6* Caspase-6, *CAT* catalase, *FADS2* Fatty acid desaturase-2, *LPL* Lipoprotein lipase, *SOD1* Superoxide dismutase-1, *SREBF2* Sterol regulatory element binding transcription factor-2, *ACTB* Beta-actin, *GAPDH* Glyceraldehyde-3-phosphate dehydrogenase

The results of differential gene abundance analysis for each comparison are provided in the Additional file 1. The analysis showed no differences in gene abundances related to lipid metabolism (lipoprotein lipase and sterol regulatory element binding transcription factor) among the dietary groups, as demonstrated by principal component analysis (Fig. 1). However, the cFF6.25 group showed distinct abundance patterns in heat shock protein and caspase-6 with respect to the control and cFF12.5 groups, as illustrated in the volcano plot (Fig. 2). Specifically, the cFF6.25 group exhibited a down regulation (*P* = 0.008) of 2 genes (heat shock protein and caspase-6) related to stress and the immune system.

## Discussion

Defining nutritional and functional proprieties of cFF intended for animal nutrition is essential for standardising practices for their efficient use [12]. The literature contains limited in-depth research on the use of cFF in poultry nutrition. Hence, the aim of this experiment was to contribute towards filling this gap. One potential challenge associated with incorporating cFF into livestock diets is its variable composition, given that it consists of a mixture of diverse raw materials. However, advancements made to former foodstuffs processors in the feed industry mean that variations in cFF composition can now be predicted by considering the different types and relative quantities of raw materials involved. This experiment diverges from the existing literature through its use of a cFF ingredient with a standardized composition formula. It responds to the gap in the literature highlighted by Luciano et al. [8], who emphasized the significant challenge in formulating a standardized base feed using former foodstuffs.

EFFPA [13] illustrated similarities among the chemical composition of cFF and common cereals, whereas in vitro studies of pig nutrition [6] indicated cFF to have a higher glycaemic index potential than corn and heat-processed wheat. Dietary starch is one of the major energy sources for monogastric species, and effective starch digestion has a significant impact on the animals’ energy status [6]. Therefore, cFF has the potential to replace other energy-rich ingredients traditionally used in feed formulations, with positive effects in terms of the circularity of food production [9].

The findings of this experiment demonstrated that inclusion of different levels of cFF in the broiler diet was compatible with achieving growth performance in Ross-308 broilers when comparing cFF fed groups with the control group. While there were no differences in the BW among dietary groups, an improvement in G:F was observed in the groups fed cFF during the grower phase as well as across the overall experimental period compared with the control group. This improvement arises from differences observed in feed consumption among groups, while ADG resulted unaffected by the dietary groups. Our results align with studies [35, 36] where high levels of dried bakery products, a type of former foodstuff, replaced corn and soybean meal in the broiler diet. Results of these experiments demonstrated that diets containing 25% or 30% dried bakery products had no adverse effects on growth performance compared with the control diet [35]. However, Potter et al. [37] observed a decrease in feed intake in turkeys fed a diet composed of 10% dried bakery products. The differences in the literature could be related to the ingredients contributing to the cFF and its chemical composition, or other aspects related to its processing methods.

Indeed, nutrient absorption can also be influenced by feed composition and the processing methods used. Ingredients like cereal flours, eggs, sugar and fats are typically mixed with water to create a dough or batter, which is then subjected to numerous technological processes that can improve their digestibility [11]. Cooking or thermal processing can alter the chemical and physical properties of food, impacting the access to and bioavailability of both macro- and micronutrients. However, the differences in growth performance observed between this experiment and the cited articles [35, 36] could also be due to the differences in the chemical composition of the diet formulations, particularly in starch content and its digestibility [38]. The experiment by Abdollahi et al. [39] included feed processing variables in the broad spectrum of the factors able to affect feed intake. Svihus [40] stated that the much higher capacity of chickens to digest the starch component of feed ingredients (even native starch) compared with other species, such as pigs, rats, and humans, may be due to their particularly abundant secretion of amylolytic enzymes in the pancreatic juice. The author also mentioned that feed intake may be inversely correlated with starch digestibility [40].

In this experiment, the inclusion of increasing levels of cFF correlated with an increase in the ATTD of DM. This finding might be attributed to the balance between simple sugars and starch in the diet, which is related to the thermal processing of cFF, as reported by Luciano et al. [11]. Additionally, factors such as fatty acid chain length and degree of saturation can influence fat digestibility in poultry diets [41]. In this experiment, the ATTD of EE could have been influenced by the reduction in levels of soybean oil in the diet, which was substituted by cFF. This reduction, along with the dietary amount of EE and unsaturated fatty acids, decreased as the levels of cFF increased. However, the ATTD of apparent metabolizable energy, gross energy, and the digestibility of amino acids were not tested and require further investigation, which represents a limitation of this experiment.

The assessment of hematological parameters provides a convenient way to evaluate the nutritional and health status of animals over the course of a feeding experiment [38]. In this experiment, cFF inclusion increased the heterophil to lymphocyte ratio, which serves an indicator of the chicken’s immunological condition, inflammation status, and stress level, which are often attributed to dietary factors [42]. This experiment is the first to assess the effects of the dietary inclusion of cFF on the hematological traits of broilers. As dietary sugar levels increase with the inclusion of cFF, we might expect an increased heterophil to lymphocyte ratio, since a diet rich in simple carbohydrates is known to promote pro-inflammatory responses, as stated by Fajstova et al. [43]. Moreover, since cFF are primarily intended for human consumption, it is possible to speculate that the presence of gluten in cFF may have enhanced the inflammatory profile of serum, given the well-known pro-inflammatory action of gluten [44]. However, the gluten level of cFF was not assessed in this experiment. Additional studies on gut microbiota and histomorphology are needed to confirm the extent of this inflammatory condition in the birds fed diets incorporating cFF.

Serum analysis also indicated an impact of the cFF diet on serum lipidic metabolites. The cFF25-fed group exhibited the highest values compared with the control diet, consistent with previous findings linking diets rich in saturated fats to elevated blood cholesterol levels [45, 46]. According to Velasco et al. [47], the source of dietary fat can impact the lipid profile of serum. In general, fats with a high concentration of SFA were found to elevate blood triacylglycerol levels, although the experiment also observed variations in the serum lipid concentrations in chickens which depended on the degree to which the sources of dietary fat were saturated.

The incorporation of cFF in the broiler diet also resulted in a noteworthy elevation of serum mineral concentrations, including phosphorus, iron, and chloride. The observed alterations may be linked to specific properties of the constituent components of cFF, which may have been subjected to mineral fortification, including high-salt products [48]. Whereas no differences in hepatic activity were revealed among dietary groups, as confirmed by aspartate aminotransferase and gamma-glutamyl transferase concentrations, the inclusion of cFF did lead to an increase in alanine-aminotransferase concentrations, which were nonetheless within the physiological range for broilers [49]. Importantly, no differences were noted in renal metabolites, with the results for creatinine indicating cFF inclusion to have no effect on kidney function in broilers. However, whereas the analysis of gene abundance related to lipid metabolism revealed no effect of the cFF diet on gene abundance at the hepatic level, 2 key genes involved in the stress response and the immune system were highlighted, namely heat shock protein and caspase-6. Under conditions of stress, heat shock protein gene abundance increases, playing a crucial role in protecting the body from oxidative stress [50]. Existing literature [50] suggests that the dietary intake of nutrients characterised by antioxidant properties might decrease the heat shock protein gene abundance. Additionally, elevated levels of caspase-6 gene have been associated with liver damage. Therefore, the lower abundance levels of these genes observed in cFF6.25-fed chickens may indicate an antioxidant activity of the ingredient and a subsequent reduction in liver stress [50]. Further investigations will be necessary to explore this possibility in depth.

## Conclusions

To the best of the authors’ knowledge, this is the first experiment that has been conducted to perform a detailed evaluation of growth performance, nutrient digestibility, hematobiochemical profiles and liver gene abundance with the inclusion of cFF (up to 25%) in the broiler diets. The results demonstrated that, although BW and ADG exhibited no differences among experimental groups, a notable increase in G:F was recorded, providing insights into the potential for cFF to become an alternative ingredient in poultry nutrition. The observed increase in ATTD of DM with increasing substitution level concur with the increase in G:F, indicating a higher digestibility of feeds containing cFF, probably due to the higher level of processing of the raw materials, originally intended for human consumption.

The differences in the serum lipid profiles between control group birds and those receiving the cFF ingredient are also worth noting. Although all values remained within the physiological ranges for broilers, the changes associated with cFF inclusion in the diet should be taken into careful consideration. It is known that industrial baked sweets often contain considerable amounts of SFA and MUFA derived from butter or margarine. Therefore, the specific selection of raw materials, choosing from both sweet and savoury varieties, may modify the fatty acid profile of cFF. The composition of cFF should be carefully formulated to make it compatible with chicken metabolism and nutrition, thus avoiding adverse changes in the blood lipid profile which could affect liver metabolism and meat quality.

Further studies are essential to validate the hematological trait findings. Nonetheless, the findings presented here suggest that the incorporation of cFF into nutritionally balanced diets, even at levels as high as 25%, does not adversely impact the overall growth performance of male broiler chickens raised until 33 days of age under commercial conditions.

## Supplementary Information

**Additional file 1: Table S1.** Effect of the different levels of cFF in broiler diets on the liver gene abundance.

## Data Availability

The analysed data from this experiment are available upon request from the corresponding author.

## Abbreviations

ADG: Average daily gain
ADFI: Average daily feed intake
ATTD: Apparent total tract digestibility
BW: Body weight
cDNA: Complementary deoxyribonucleic acid
cFF: Commercially processed former foodstuffs
CP: Crude protein
DM: Dry matter
EE: Ether extract
G:F: Gain to feed ratio
MUFA: Monounsaturated fatty acids
PUFA: Polyunsaturated fatty acids
RNA: Ribonucleic acid
SEM: Standard error of the mean
SFA: Saturated fatty acids
TiO_2_: Titanium dioxide
