# Supplementary material for: Dietary processed former foodstuffs for broilers: impacts on growth performance, digestibility, hematobiochemical profiles and liver gene abundance
Source: J Anim Sci Biotechnol. 2024 Sep 8;15:122. doi: 10.1186/s40104-024-01081-w (PMC11380770; doi:10.1186/s40104-024-01081-w)
Supplement: Supplementary file 1 — Additional file 1: Table S1. Effect of the different levels of cFF in broiler diets on the liver gene abundance. [file 40104_2024_1081_MOESM1_ESM.docx]

**Differential gene abundance analysis results**

**Table S1** Effect of the different levels of cFF in broiler diets on the liver gene abundance (*n* = 5 per dietary group)

| **Genes** | **cFF25 vs. cFF0** | | **cFF6.25 vs. cFF0** | | **cFF12.5 vs. cFF0** | | **cFF12.5 vs. cFF25** | | **cFF6.25 vs. cFF12.5** | | **cFF6.25 vs. cFF25** | |
| --- | --- | --- | --- | --- | --- | --- | --- | --- | --- | --- | --- | --- |
|  | **log2 (Fold Change)** | ***P*_adj._  value** | **log2  (Fold Change)** | ***P*_adj._  value** | **log2  (Fold Change)** | ***P*_adj._  value** | **log2  (Fold Change)** | ***P*_adj._  value** | **log2  (Fold Change)** | ***P*_adj._  value** | **log2  (Fold Change)** | ***P*_adj._  value** |
| *ACOX1* | 0.505 | 0.783 | 0.881 | 0.330 | 0.379 | 1.000 | -0.126 | 0.953 | 0.501 | 0.722 | 0.376 | 0.927 |
| *FABP1* | 0.399 | 0.801 | 0.627 | 0.687 | -1.140 | 0.715 | -1.539 | 0.525 | 1.767 | 0.225 | 0.228 | 0.927 |
| *HSPA2* | -1.554 | 0.432 | -2.564 | 0.008 | -0.163 | 1.000 | 1.391 | 0.525 | -2.401 | 0.012 | -1.010 | 0.540 |
| *CASP6* | -1.182 | 0.432 | -2.341 | 0.008 | 0.306 | 1.000 | 1.488 | 0.525 | -2.647 | 0.003 | -1.159 | 0.480 |
| *CAT* | -0.448 | 0.801 | 1.534 | 0.330 | 1.089 | 0.715 | 1.537 | 0.525 | 0.445 | 0.752 | 1.982 | 0.480 |
| *FADS2* | 1.607 | 0.432 | 1.920 | 0.180 | 2.572 | 0.081 | 0.964 | 0.598 | -0.652 | 0.722 | 0.313 | 0.927 |
| *LPL* | 0.776 | 0.783 | 0.551 | 0.687 | 0.983 | 0.715 | 0.206 | 0.953 | -0.432 | 0.722 | -0.225 | 0.927 |
| *SOD1* | 0.416 | 0.783 | 0.642 | 0.330 | -0.097 | 1.000 | -0.513 | 0.598 | 0.739 | 0.249 | 0.226 | 0.927 |
| *SREBF2* | 0.917 | 0.432 | 0.055 | 1.000 | 1.487 | 0.081 | 0.571 | 0.598 | -1.433 | 0.044 | -0.862 | 0.480 |
| *ACTB* | -0.379 | 0.801 | -2.393 | 0.005 | -0.049 | 1.000 | 0.330 | 0.894 | -2.345 | 0.003 | -2.015 | 0.062 |
| *GAPDH* | 0.000 | 1.000 | 0.000 | 1.000 | 0.000 | 1.000 | 0.000 | 1.000 | 0.000 | 1.000 | 0.000 | 1.000 |

*P_adj._,* adjusted *P*-value*; ACOX1,* acyl-CoA oxidase 1*; FABP1,* fatty acid binding protein 1*; HSPA2,* heat shock protein*; CASP6,* caspase 6 *; CAT,* catalase*; FADS2,* fatty acid desaturase 2*; LPL,* lipoprotein lipase*; SOD1,* superoxide dismutase 1*; SREBF2,* sterol regulatory element binding transcription factor 2*; ACTB,* Beta-actin*; GAPDH,* glyceraldehyde-3-phosphate dehydrogenase*; cFF,* commercially processed former foodstuffs*; cFF0*, control diet (based on corn, soybean meal and soybean oil); *cFF6.25*, 6.25% w/w substitution of corn, soybean meal and soybean oil with cFF; *cFF12.5*, 12.5% w/w substitution of corn, soybean meal and soybean oil with cFF; *cFF25*, 25% w/w substitution of corn, soybean meal and soybean oil with cFF
